# Regulation of Ovarian Cancer Prognosis by Immune Cells in the Tumor Microenvironment

**DOI:** 10.3390/cancers10090302

**Published:** 2018-09-01

**Authors:** Maureen L. Drakes, Patrick J. Stiff

**Affiliations:** Cardinal Bernardin Cancer Center, Department of Medicine, Loyola University Chicago, Building 112, 2160 South First Avenue, Maywood, IL 60153, USA; pstiff@lumc.edu

**Keywords:** tumor microenvironment, immune inhibition, tumor-infiltrating lymphocytes, tumor-associated macrophages, dendritic cells, antitumor immunity, immunotherapy

## Abstract

It is estimated that in the United States in 2018 there will be 22,240 new cases of ovarian cancer and 14,070 deaths due to this malignancy. The most common subgroup of this disease is high-grade serous ovarian cancer (HGSOC), which is known for its aggressiveness, high recurrence rate, metastasis to other sites, and the development of resistance to conventional therapy. It is important to understand the ovarian cancer tumor microenvironment (TME) from the viewpoint of the function of pre-existing immune cells, as immunocompetent cells are crucial to mounting robust antitumor responses to prevent visible tumor lesions, disease progression, or recurrence. Networks consisting of innate and adaptive immune cells, metabolic pathways, intracellular signaling molecules, and a vast array of soluble factors, shape the pathogenic nature of the TME and are useful prognostic indicators of responses to conventional therapy and immunotherapy, and subsequent survival rates. This review highlights key immune cells and soluble molecules in the TME of ovarian cancer, which are important in the development of effective antitumor immunity, as well as those that impair effector T cell activity. A more insightful knowledge of the HGSOC TME will reveal potential immune biomarkers to aid in the early detection of this disease, as well as biomarkers that may be targeted to advance the design of novel therapies that induce potent antitumor immunity and survival benefit.

## 1. Introduction

Ovarian cancer may be divided into six subgroups, namely, serous, mucinous, endometroid, transitional-cell, clear-cell, and squamous carcinoma [1]. The most common group is high-grade serous ovarian carcinoma (HGSOC), a disease that escapes detection and diagnosis until after it is disseminated to areas of the abdomen and beyond. At this advanced stage, survival is dismal, with only about twenty percent of patients diagnosed at International Federation of Gynecology and Obstetrics (FIGO) stage III or IV disease fortunate enough to reach a five-year survival time point, since most of these individuals become resistant to conventional therapy and succumb to disease. This disease grows aggressively, often recurs at the primary or metastatic sites, and is the most deadly of gynecologic cancers [2,3].

HGSOC is believed to arise from the ovarian-surface epithelium and/or the fallopian epithelium [4]. Most patients (96%) with this disease have TP53 mutations, with BRCA1/2 (22% patients) mutations also common [5,6]. An accompanying feature of HGSOC is an accumulation of ascites fluid in the peritoneal cavity, which allows the adhesion of cancer cells to the omentum (connective and fatty tissue covering the ventral surface of the intestines) and serous membranes lining the peritoneal organs [7], thereby increasing the potential of cancer lesions at these sites soon after the primary disease is established [8,9].

The tumor microenvironment (TME) in HGSOC is comprised of an intricate system of immune cells, including subsets of T cells, dendritic cells, macrophages, and NK cells, as well as soluble factors elaborated by myriads of existing cell types, both spontaneously and as a result of their networking interactions [10,11]. Studies on the ovarian TME in HGSOC have been prompted by the need to understand the disease biology, with the goal of targeting cancer-promoting immune mechanisms, and providing effective therapies for the management and ultimately a cure for HGSOC. The full significance of the ovarian TME in determining disease progression, recurrence, or regression is yet to be revealed. This review focuses on the dynamic and diverse immune components in the ovarian TME and how they mediate the balance between protumor and antitumor immunity, and patient survival.

## 2. Immune Regulation by T Cells in the TME

Tumor-associated/infiltrated lymphocytes (TILs) are found in the tumor stroma or in the tumor islets (intraepithelial TILs). CD3+, CD4+, and CD8+ TILs are usually associated with a positive outcome [12,13,14,15,16]. Notably, in a study of 186 samples of advanced-stage ovarian cancer, it was found that 55% patients with CD3+ TILs had a five-year survival of 38%, whereas only 4.5% patients without detectable TILs reached his survival mark. Moreover, taking into consideration patients who had surgical debulking and platinum-based chemotherapy, 73.9% of patients with pre-existing TILs had complete response (CR), while only 11.9% patients without TILs exhibited CR [15].

Some groups have demonstrated that in ovarian-cancer tumor-tissue sections, intraepithelial CD8+ TILs correlate with good outcome, and others have shown that a high ratio of CD8+/FoxP3+ T regulatory cells (Tregs) is beneficial to survival [17]. In a meta-analysis of 10 studies and 1815 patients, both CD3 and CD8 TILS were found to be associated with survival, but CD8+ TILS were the more significant of these two subsets. Interestingly, in these studies the prognostic value of TILS was more significant in some geographic regions studied compared with others, raising the possibility that genetic factors or different levels of access to healthcare may also be relevant factors to consider when measuring survival in such studies [13].

In detailed investigations with over 5500 patients, including 3196 with HGSOC, it was found that among the five invasive histotypes studied, HGSOC showed the most infiltration of CD8+ T cells. Patients were followed over 24,650 person-years. Analysis of CD8+ TILS in the tumor epithelium on a scale of negative, low, moderate, and high revealed distinct survival differences in HGSOC patients based on the density of CD8+ TILS in the epithelial components of tumor islets. The median survival for patients with no CD8+ TILS was 2.8 years, whereas with low, moderate, or high TILS, survival was 3.0 years, 3.8 years, and 5.1 years, respectively. The presence of CD8+ TILS was favorable to outcome regardless of extent of residual disease, standard therapy or BRCA1 mutation [18].

Others report that the CD8+CD103+ T cell subset are found in abundance in the ovarian-cancer epithelium, and are associated with a better outcome [19,20]. Together, these studies of CD8+ T cells in the ovarian TME further emphasize the relevance of these TILS as a prognostic indicator in HGSOC.

**Figure 1:**
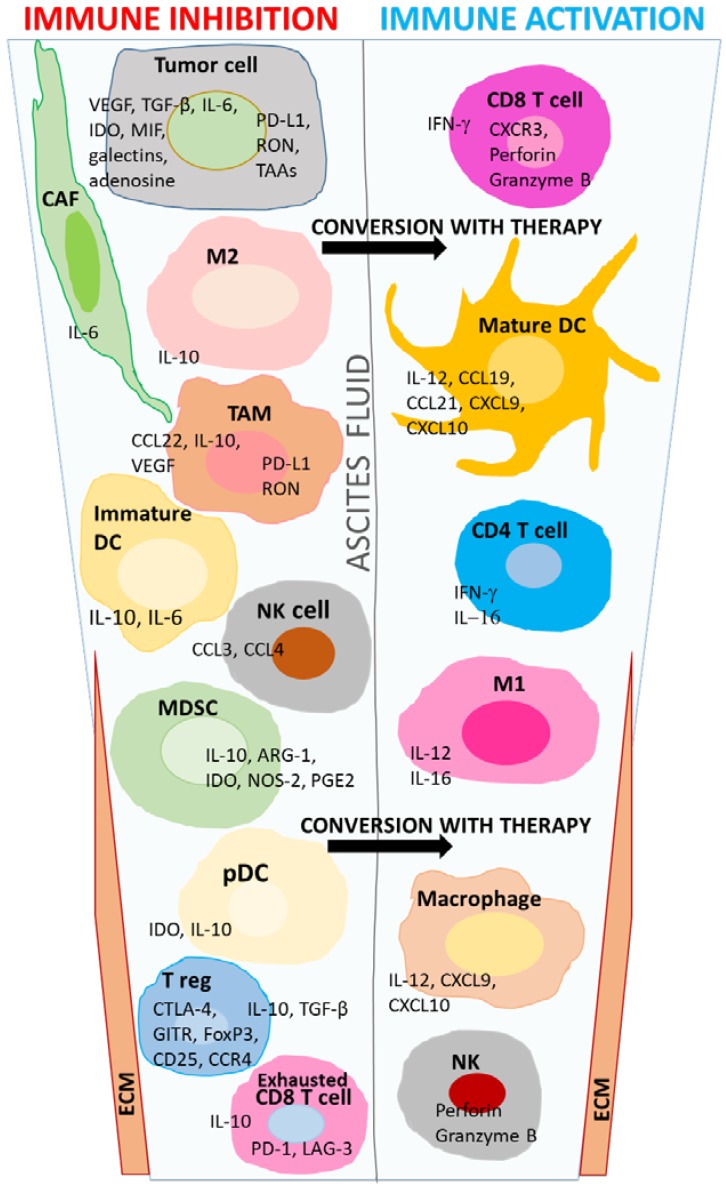
Schematic representation of the primary immune components in the tumor microenvironment (TME). Several cell types in the TME of high-grade serous ovarian carcinoma (HGSOC) elaborate factors that can lead to immune dysregulation and inhibition of antitumor responses. The ascites of these patients is rich in TGF-β, IL-6, IL-8, IL-10, vascular endothelial growth factor (VEGF), and CCL22 and other factors released by contributing cell types as shown in the graphic. CCL22 (the ligand for CCR4) preferentially recruits Tregs into tumors. Exhausted CD8 T cells in tumors express PD-1 and LAG-3 and secrete low quantities of IFN-γ. Several Treg subsets exist in the TME, each bearing some of the phenotypic markers, CD4, CD8, CCR4, FoxP3, CD25, GITR, or CTLA-4, and primarily release TGF-β and IL-10. Molecules such as recepteur d’origine nantais (RON) on tumor cells are associated with invasiveness, and tumor associated antigens (TAAs) such as New York Esophageal antigen-1 (NY-ESO-1), human epidermal growth factor receptor 2 (HER-2), and Wilm’s tumor-1 (WT-1) are immunogenic targets. Immune-suppressive mechanisms in the TME that foster tumor initiation, progression, and recurrence may be reversed with combinations of conventional and novel therapies, designed to potentiate antitumor immune responses. Parameters consistent with disease improvement include CD8+ T cells secreting IFN-γ, perforin, and granzyme B, which facilitate the killing of tumor cells. Additionally, DC-secreted chemokines, such as CXCL9 and CXCL10, can recruit CD4+ and CD8+ immunocompetent T cells, and IL-16-a-cytokine secreted by T cells, macrophages, and dendritic cells, is a primary chemoattractant for CD4+T cells in ovarian cancer.

Another subset of TILs populating the HGSOC TME are FoxP3+ T regulatory cells. These cells were initially regarded as a potent immunosuppressive mechanism, limiting the potency of antitumor immune responses. CD4+CD25+FoxP3+ T regulatory cells may act by elaborating protumor cytokines such as IL-10 or TGF-β, or by cell–cell contact mechanisms [21]. Despite several early reports associating this subset of T regulatory cells with a poor outcome [22,23], a meta-analysis of 869 patients over several studies did not conclude that FoxP3 Tregs in the tumors of ovarian-cancer patients are a significant prognostic indicator of survival [24]. Yet there are other T cell subsets in the ovarian TME that may negatively impact survival. These include T cells expressing cytotoxic T lymphocyte-associated antigen 4 (CTLA-4), glucocorticoid-induced TNF receptor family-related protein (GITR), or CCR4, CD8+CD28- T regs [25,26,27,28], as well as exhausted CD8+T cells expressing immune checkpoint inhibitory molecules programmed cell death-1 (PD-1) or lymphocyte activation gene-3 (LAG-3; CD223) [29,30,31]. These cells may all confer immunosuppression in the TME or limit antitumor responses (Figure 1). There is still debate in the literature concerning the role of the Th17 CD4+ T cell subset in ovarian cancer, but some have reported that these cells have an inverse relationship with Tregs, and correlate with survival [32,33].

In addition to their prime role in immune surveillance limiting the initiation of ovarian cancer and other cancers [34,35,36,37], immunocompetent TILs can recognize cancer antigens or overexpressed self-antigens that have been processed by antigen-presenting cells, and mount potent antitumor immune responses. CD4+ TILs can recruit dendritic cells that can prime T cells to exert their cytotoxic effects by secreting perforin, granzyme B, or Fas ligand (cell death receptor ligand; FasL; CD95L), which may directly kill cancer cells. Both cytotoxic CD8+ and CD4+ TILs cells secrete cytokines such as IFN-γ and IL-2 [38] that can induce other cells in the TME to mount antitumor immunity, and promote longer survival. In the ovarian tumor, IL-16, primarily a Th1 cytokine, has been reported to be a critical chemoattractant for the recruitment of CD4+ T cells into the tumor [39].

However, there is an ongoing interplay between TILs and the TME, and by suppressing the function and limiting the infiltration of CD3+, CD4+, or CD8+ TILs, tumors can circumvent antitumor immunity, especially in TME where TILs were already low in numbers at the time of diagnosis. Exclusion or inhibitory mechanisms imposed on TILs in the ovarian TME are as follows. Increased angiogenesis in ovarian-cancer cells presents a great barrier to the infiltration of tumor-specific T cells, thereby reducing the numbers of TILs in patients’ tumors. TGF-β increases angiogenesis directly as well as suppresses the proliferation and activation of TILs [40,41]. Overexpressed vascular endothelial growth factor (VEGF) can enhance the proliferation, migration, and invasion of endothelial cells and is associated with poor outcomes in ovarian cancer [42]. VEGF-A decreases the adhesive interaction between lymphocytes and tumor vascular endothelial cells, and reduces TIL penetration through deregulation of intercellular adhesion molecule-1 (ICAM-1) and vascular cell adhesion-molecule-1 (VCAM-1) [43]. Furthermore, VEGF-A in concert with IL-10 and PGE2 induces FasL in endothelial cells. Increased FasL in endothelial cells favors the selective trafficking of Tregs above CD8+ immunocompetent T cells [44]. Several other molecules in the tumor endothelium, such as programmed cell death-1 ligand (PD-L1), B7-H3, arginase-1 (ARG-1), indoleamine 2,3, dioxygenase (IDO), IL-10, and PGE2, released by endothelial cells, downregulate TIL function or kill CD8+ effector TILs [45,46,47,48,49].

The density of TILs in tumors has recently been used to categorize tumors. Tumors are termed “hot” or immunogenic if they consist of high numbers of TILs, whereas “cold tumors” have much fewer TILs [50,51], and patients in this latter group are likely to have a poor response to therapy. An understanding of T cell subsets in relation to inhibitory mechanisms in the ovarian TME at baseline diagnosis is crucial to effectively designing novel therapies for HGSOC, and to predict outcome after treatment regimens, as T cells (especially CD8+ T cells) may be the critical antitumor effector mechanism in the disease. The success of these therapies may depend largely on the ability of T cells to reverse immune dysregulation at the site of the disease.

## 3. Multifaceted Nature of Macrophages in the TME

In contrast to immunocompetent T cells, the majority of myeloid lineage cells in the ovarian TME are generally of a protumor propensity [52,53]. Subpopulations of myeloid lineage cells in the TME of patients consist of a variety of phenotypes and nomenclature [54,55] (Table 1).

Tumor-associated macrophages (TAMS) are the major subpopulation of this lineage cells in the ovarian TME. TAMS can readily change phenotype and function in the presence of soluble molecules in the surrounding milieu [56,57]. These cells can be recruited from blood monocytes, or arise from resident peritoneal macrophages [54,58,59,60]. TAMS from both of these origins have some phenotypes in common such as the expression of molecules CD163 and CD206, as well as similar levels of genes for phagocytosis and antigen presentation. However, a distinctive feature of TAMS in the TME is an upregulation of genes linked to extracellular-matrix (ECM) remodeling [61]. In ovarian cancer, TAMS are mostly immunosuppressive, and associated with tumor cell invasion, angiogenesis, metastasis and early relapse [11,62,63].

In tumors, the benign-to-malignant state is associated with angiogenesis (increase in vascularization). VEGF, TGF-β, matrix metalloproteinases (MMPs), hypoxia-inducible factor (HIF), and adrenomedullin (ADM) secreted by TAMS enhance the process of vascularization [64,65,66]. TAMS are also critical in mediating epithelial–mesenchymal transition (EMT), which is essential to tumor progression. In this process, polarized epithelial cells change their phenotype to motile mesenchymal cells. The downregulation of epithelial markers, such as E-cadherin, is replaced by the upregulation of mesenchymal markers such as vimentin, Slug, Snail, fibronectin, zinc-finger E-box binding homeobox 1 (ZEB1), ZEB2, and α-smooth muscle actin, allowing cells to migrate and invade [61,66,67]. These changes correlate with metastasis, recurrence, chemoresistant tumors, and poor outcome. EMT is mediated by several TAM products, such as TGF-β, hepatocyte growth factor (HGF), and epidermal growth factor (EGF) [66,68,69,70,71].

Studies in ovarian-cancer tissue showed that there is a significant elevation in the numbers of CD68+ and CD206+ TAMS, and of MMPs expression, in comparison with benign ovarian tissues [72]. This difference was due to higher levels of these parameters in patients with stage III/IV in comparison with those at stage I/II disease. Furthermore, in patients with positive lymphatic invasion, the numbers of CD68+, CD206+, and MMP-positive cells was significantly higher than in patients without lymphatic invasion [72]. Additional studies with SKOV3 ovarian-tumor cells demonstrated that TAMS promoted upregulation of TLRs 1, 2, 4 and 6, MMP-2, MMP-9, and MMP-10 expression. Ovarian-cancer cell invasion was enhanced via TLRs signaling pathway and activation of downstream nuclear transcription factor (NF)-KB p65 and microtubule-associated proteins (MAPs) kinases pathway in SKOV3 cells [72].

In other studies, TAMS enhanced spheroid formation and tumor growth and early ovarian-cancer metastasis by secreting EGF [62]. TAMS can also be high secretors of CCL18, a chemokine that promotes tumor migration and metastasis in ovarian cancer [73]. IFN-γ treatment reduces CCL18 secretion and can switch TAMS to an immunostimulatory phenotype [74]. TAMS in the ovarian-cancer TME are very low IL-12 secreting, a cytokine that is positively associated with outcome in this disease [61,75,76]. A brief summary of the primary protumor processes regulated by TAMS in the TME is outlined in Table 2. A more detailed account of TAM activities in the TME is reported elsewhere [66,70].

Macrophages and monocytes in the ovarian tumor may exhibit polarization to an M2, protumor, and immunosuppressive state, under the influence of colony-stimulating factor (CSF), IL-4, IL-13, IL-10, TGF-β and other soluble molecules. M2 (alternatively activated) macrophages secrete IL-10 and TGF-β and play an active role in tissue remodeling and tumor progression [77,78].

The presence of CD4+ or CD8+ T cells secreting IFN-γ in the tumor can promote the presence of M1 immunocompetent classically activated macrophages, above an M2 phenotype. M1 macrophages are stimulated by Toll-like receptors (TLR) ligands and by IFN-γ to give a Th1 response secreting IL-12, IL-23, and TNF-α. M1 macrophages are highly potent against micro-organisms and tumors, and are associated with survival in HGSOC [79].

Another subgroup of TAMS that also merits special mention is the myeloid-derived suppressor cell (MDSC, M-MDSC). This is a group of immature myeloid cells in the TME that correlate well with heightened disease, increased tumor burden, and resistance to immune therapy in HGSOC [80,81]. These cells have a role in enhancing stemness and promoting metastasis of ovarian-cancer cells by inducing miRNA101 expression, subsequently repressing the corepressor gene C-terminal binding protein-2 (CtBP2) [82].

MDSC are also recruited to the ovarian TME under the influence of chemokine receptor CXCR4. PGE2 is required for the production of chemokine CXCL12, and for the expression of its binding receptor CXCR4 in these cells [83]. The CXCR4–CXCL12 axis and PGE2 are critical to the progression of HGSOC [84,85,86], and negatively impact the function of several immune cells in the TME, as we will discuss in the subsequent text.

Cyclooxygenase-2 (COX-2, an enzyme required for PGE2 synthesis) and PGE2 drive the differentiation of CD1a+ DC to CD14+CD33+CD34+ MDSC, and induce the expression of immunosuppressive molecules IDO, arginase-1 (ARG-1), IL-10, nitric oxide synthase-2 (NOS-2) and COX-2 by MDSC, molecules that limit CD8+ cytotoxic T cell responses [49,87,88]. Blocking COX-2/PGE2 suppression in MDSC prevents the accumulation of MDSC and enhances antitumor immunity [85]. Additional stimuli that may recruit MDSC to the ovarian TME are soluble factors such as VEGF, which are secreted by tumor cells in the microenvironment [81]. Granulocytic-MDSC (G-MDSC) are also of the myeloid lineage but do not appear to be a critical factor in the progression of most cancers.

Taken together, TAMS play critical roles in the establishment of cancers, including HGSOC. Targeting of these cells with anti-CCL2 antibody, anti-CSF-IR inhibitors, anti-CD52 antibody, and anti-CD11b antibody for therapy of ovarian cancer has been investigated in preclinical models of ovarian cancer [89,90,91]. There has also been a limited number of Phase I/II clinical trials blocking TAM activity in patients [92,93], but to date there is no such approved therapy.

## 4. Dendritic Cell Function in the Ovarian TME

Dendritic cells capture antigen, process and present antigenic peptide to cells in the immune system [94]. DC present exogenously captured peptides to CD4+ T cells via MHC class-II, and endogenous peptide antigens via major histocompatibility complex class-I (MHC-I) to CD8+ T cells. DC can also present exogenously captured antigens as MHC class I associated peptides (cross presentation), consequently facilitating more efficient CD4+ and CD8+ T cell activity [95,96]. Potent activation of T cells requires a cognate antigen (signal 1), costimulatory molecules (such as CD80, CD86, CD40) on DC or other antigen-presenting cells (signal 2), and proinflammatory cytokines (signal 3). If this process is sequential and efficient the outcome is Th1 (antitumor) immunity by CD4+ and CD8+ T cells. Lack of any of these signals can result in Th2 immunity or immune suppression mediated by Tregs [97,98,99,100,101]. Tumors can disrupt these signals by strategies such as loss of tumor antigens, and by the abundance of immunosuppressive soluble factors in the TME that can induce DC dysfunction [102,103].

Immature myeloid DC are derived from hematopoietic bone-marrow (BM) progenitor cells. These cells leave the BM enter the bloodstream and reside in lymph nodes or other tissue. They express costimulatory molecules at low levels, release low levels of cytokines, and are capable of mounting only limited immune responses. These cells express chemokine receptors CXCR3, CXCR4, CCR1, 2, 5, and 6. On stimulation by antigen, immature DC migrate to lymph nodes from tissues and present the specific antigen to other immune cells [104,105].

DC exposed to antigen undergo a process of maturation, characterized by an increase in costimulatory molecules, downregulation of existing chemokine receptors, and the acquisition of CCR7, the latter of which recruits DC to LN, attracted by CCL19 (MIP-3β) and CCL21, chemokines secreted by DC. Mature DC can activate naïve CD8+ T cells, crosslink with CD40 ligand on other cells, and secrete IL-12 [94,104,105,106,107].

Myeloid DC in tumors are found in low numbers and exhibit many features of immature DC. The immune-suppressive environment in the ovarian tumor, rich in TGF-β, IL-10, VEGF, ARG-1, along with inhibitory molecules such as IDO, PD-1, and PD-L1, drives the differentiation of CD14+CD1a- immature myeloid cells, anergic T cells and Tregs, induces tolerance, and promotes tumor growth [49,108,109]. In one study, depletion of DC in mice at advanced stages of ovarian cancer delayed tumor growth [110]. The benefit of mature myeloid DC function in inducing antitumor immune responses has been exploited in DC vaccine therapy clinical trials in ovarian and other cancers (NCT00703105) [111,112,113].

Recent evidence indicates that in melanoma, tumor-residing CD103+ DC were necessary for CD8+ effector T cell recruitment in the TME. These CD103+ DC have high expression of CXCR3 and of the transcription molecule Batf3, which possibly controls the development and maintenance of the DC1 lineage [114,115,116]. The presence of Baft3-lineage CD103+ DC correlated with the presence of CXCR3-binding chemokines CXCL9, 10 and 11, which increase the trafficking of effector T cells into tumors, and are associated with survival in cancers such as HGSOC [117,118]. The lack of conventional DC as in Batf3 −/− mice abolishes the rejection of immunogenic tumors, the response to adoptive T cell therapy, and to immune checkpoint blockade [114,115,119,120]. It is plausible that in the ovarian TME a similar mechanism of recruitment of effector T cells by this DC lineage would be an immune-enhancing mechanism to counteract the underlying immunosuppressive myeloid networks that favor disease progression, recurrence, and death.

In addition to immature myeloid-derived DC, plasmacytoid DC also contribute to the immunosuppressive network in HGSOC. CXCR4 expressing plasmacytoid DC (pDC) precursor cells are recruited into the ovarian TME by CXCL12 and IL-10 in the tumor [121]. Plasmacytoid DC (CD4+CD123+BDCA2+) in tumors such as HGSOC are often tolerogenic, and are noted for the release of IDO, an enzyme that catalyzes tryptophan degradation [47,48]. IDO promotes tumor angiogenesis and metastasis, and downregulates the proliferation and other functions of TILS [122].

In ovarian cancer, pDC induced IL-10 secreting CD4+ and CD8+ Tregs and enhanced angiogenesis, mediated by the secretion of TNF-α and IL-8. Tumor pDC produced low quantities of IL-6, TNF-α, IFN-α, macrophage inflammatory protein-1β (MIP-1β), and RANTES (CCL5) in response to TLR stimulation, in contrast to pDC from ascites or peripheral blood. In a cohort of 44 ovarian-cancer patients, pDC were the most abundant DC subset in tumor and malignant ascites, but they were almost depleted in peripheral blood. The presence of pDC in the tumor only (but not in ascites) was associated with early relapse [123].

## 5. Tumor-Associated Neutrophils

Polymorphonuclear neutrophils (PMNS, neutrophils) are of the myeloid lineage of cells and exhibit some of the phenotypes of G-MDSC (CD33+CD66b+). However, transcriptome analysis shows these cell types to be two distinct populations [124]. Neutrophils are a heterogenous group of cells that may be classified into two main functional groups, antitumor (N1) and protumor (N2) [125]. Neutrophils move into tissues from blood under the influence of CXCL1 and CXCL2 and other mediators [126,127].

The role of tumor-associated neutrophils (TANS) in the ovarian TME is not yet fully elucidated. Recent investigations showed that coincubation of ovarian-cancer SKOV3 cells with either PMNS or PMN lysate changed the polygonal epithelial phenotype of the cells to a spindle shape, causing a cribform cell growth. This PMN-induced alteration was due to elastase, a prominent protease of PMN. PMN elastase induced changes in cells were consistent with an EMT process of the cancer cells, and a more migratory phenotype. These authors also studied 213 HGSOC patient samples and showed that PMN are a significant portion of TILS in many patients. Some biopsies showed a definite clustering of PMN and ZEB1 (EMT transcription factor)-positive cancer cells, especially in areas of low E-cadherin [128]. Transition from an epithelial to mesenchymal profile is characteristic of a more aggressive nature in cancers.

In the TME, TGF-β appears to be the predominant soluble molecule responsible for tumor associated neutrophil (TAN) polarization, and inhibition of this molecule favors the accumulation of N1 TANS [125,129]. Neutrophils have a prime role in the initiation of tumors as they act to alter the ECM and the TME. MMP-9 secreted by neutrophils is a key upregulator of carcinogenesis [130]. Additional destructive roles of neutrophils in the TME, such as contributing to angiogenesis, extravasation, and metastases, and suppression of the adaptive immune response are well-reported, as summarized elsewhere [131,132,133].

N1 TANS exhibit protection against tumor development through several mechanisms. They may directly kill tumor cells, or they can promote CD8+ T cell recruitment and activation by elaborating T cell-attracting chemokines such as CXCL9 and CXCL10, and Th1 cytokines such as IL-12 [134,135,136,137]. Several other mechanisms whereby N1 TANS potentiate antitumor immunity have also been reported [133,138].

The prognostic value of neutrophils in ovarian cancer is further underscored by the findings of a recent meta-analysis study, which showed that a high neutrophil-to-lymphocyte ratio (NLR) was associated with worse overall survival (O/S) in some groups of patients (Asians, but not in Caucasians) [139].

## 6. Natural Killer Cells

Natural killer (NK) cells are an integral part of the innate immune system. These cells do not rely on HLA-mediated recognition of tumor targets, rather, the CD16 receptor, the NKG2D receptor and the NKp30 cytotoxicity receptor on NK cells mediate the death of tumor cells. CD56 high CD16- NK cells have low cytotoxic potential, whereas CD56 low CD16+ NK cells are more efficient at killing tumor cells. In ovarian cancer, there may be defects in NK-cell function such as aberrant receptor or ligand expression, fewer NK cells, or inability of these cells to effectively secrete cytotoxicity molecules or cytokines, which are all possible mechanisms of immune escape [58]. For example, cancer cells from ovarian cancer ascites fluid release macrophage migration inhibitory factor (MIF), a chemokine that stimulates tumor-cell proliferation, migration, and metastasis. MIF transcriptionally downregulates NKG2D in NK cells and lowers the ability of these cells to kill tumor cells [140]. Additionally, high expression of soluble B7-H6 (a ligand for the NKp30 receptor) was associated with lowered NKp30 expression on NK cells and reduced NK-cell activity [141]. It has also been reported that lower B7-H6 expression correlates with reduced metastasis and disease progression, and better overall survival in ovarian cancer [142].

In the presence of IL-18, NK cells can release chemokines CCL3 and CCL4, which attract immature DC. Efficient NK–DC interaction in the tumor can lead to increase of CXCR3 and CCR5 on DC, which can recruit CD8+ effector T cells to tumors, in the presence of chemokines CXCL9, CXCL10, and CCL5 [143]. Gene-analysis expression from the Immunological Genome Project showed that NK cells can secrete CCL5, CCL3, XCL1, CXCL1, CCL4, and CCL27A [144,145]. In tumors, NK cells were strong inducers of conventional DC chemoattractants XCL1 and CCL5. Tumor production of PGE2 could disrupt this process and the ability of DC to secrete chemokines [145]. Taken together, NK cells can directly regulate tumor-cell numbers through cytotoxic mechanisms, or NK cells can potentiate the efficacy of antitumor T cell responses through adaptive immune mechanisms.

Investigations have been conducted using an IL-15 superagonist complex, IL-15N72D/IL-15Rα-Fc (ALT-803; Altor Bioscience Corporation, FL, USA), which inhibits complement activation, and includes the addition of a domain to mediate IL-15/IL-15Rα transpresentation to NK cells. In this study, NOD/SCID/γc−/− (NSG, which do not contain NK/NKT/**γδ** T/B cells) mice were xenografted with firefly luciferase-expressing MA148 tumor cells, and sublethally irradiated. Mice were then administered overnight activated human NK cells, followed by ALT-803, and analyzed for tumor cells at different time points. When mice were euthanized, a peritoneal lavage was performed and NK-cell function evaluated [146].

Mice treated with ALT-803 resulted in an NK-dependent significant decrease in tumor. ALT-803 also enhanced the cytotoxic function (as measured by increases in CD107a, IFN-γ, and TNF-α) of NK cells from PBMC or ascites, when coincubated with ovarian-cancer cell lines [146]. Targeting of NK cells in a clinical setting may be a promising therapy strategy in HGSOC.

## 7. Other Components of the Ovarian TME

### 7.1. TME Architecture

An underlying factor in metastasis involves the attachment of ovarian-cancer cells in ascites to areas of the abdomen. The mesothelium, the squamous epithelium that covers organs of the peritoneal cavity, consists of a single layer of mesothelial cells, below which is a basement membrane of collagen, fibronectin, and laminin, components of the ECM. Some studies showed that cancer cells from ascites preferentially attach to the basement membrane rather than to mesothelial cells [147], suggesting that this mesothelial layer may be a limited frontline defence against ovarian-cancer progression. However, it is also known that ovarian-cancer cells also directly attach to mesothelial cells via β1 integrin and CD44 [71,148,149,150]. During this process, ovarian-cancer cells upregulate mesenchymal genes such as *TWIST1* and *ZEB1* [149], and decrease the expression of genes such as *CDH1*, an epithelial gene for E-cadherin [71]. There are several other processes whereby ovarian-cancer cells may invade the mesothelial cell layer, such as by actively killing mesothelial cells. In colon-cancer cells for example, a Fas (expressed on mesothelial cell)- Fas ligand (expressed on cancer cells) mediated mechanism of killing mesothelial cells has been described [150].

As earlier addressed, TAMS also play a central role in altering the ECM, thereby contributing to the adhesion, invasion, and proliferation of ovarian-cancer cells. Additionally, adipocytes of the omentum contribute to a protumor TME by secreting IL-6, IL-8, CCL2, and adiponectin, which support ovarian-cancer cell metastasis [151].

Cancer-associated fibroblasts (CAFs) contribute to excessive deposition and alteration of the ECM, creating a barrier that blocks efficient delivery of anticancer drugs and enhancing chemoresistance [152]. CAFs also secrete a range of protumor molecules that create an immunosuppressive milieu in the ovarian TME, and support the proliferation, invasion, and migration of cancer cells [153,154,155,156,157]. In an epithelial ovarian-cancer (EOC) xenograft model, human bone-marrow mesenchymal stem cells were shown to give rise to CAFs that produced IL-6 to enhance tumor growth [158].

### 7.2. Exosomal Vesicles (EVs)

These vesicles are released by tumor cells and most other cells types of the TME [159,160]. They mediate the transfer of proteins, lipids, and nucleic acids such as DNAs, mRNAs, and miRNAs between tumor and stroma [161]. EVs range from 30 to 150 nm, whereas microvesicular bodies (MVBs) are 100 nm to 1 µm [162]. EVs carry molecules such as CD24, and epithelial cell adhesion molecule (EPCAM1), which directly regulate cancer-cell migration, proteases (MMP2, MMP9), which promote ECM degradation and cancer invasiveness [160,163,164], or EV-associated mRNAs, such as miR21, which may induce resistance to paclitaxel [163,165,166].

## 8. Interactive Communication in the TME

Characteristics of HGSOC are aggressive growth and recurrence of tumors within the peritoneal cavity as well as metastasis to other sites. Novel therapy to manage ovarian cancer is tailored to overcome immune suppressive mechanisms in the TME that contribute to reduced immune surveillance and immune evasion by tumor cells. Since the TME in each HGSOC patient is both heterogenous and unique [167], there is the need for a better understanding of the contribution of the TME to disease outcome, and more adequate tools to evaluate patients in this present era of personalized therapy.

Blank and colleagues [168] proposed an immunogram model, consisting of seven parameters, which describes interactions between cancers and the immune system that may occur in individual patients. In this framework, the assumption is that T cell activity is the ultimate effector mechanism in therapy response, and that even though other cells, or other factors such as modulation of the microbiome, may contribute to outcome, the contribution to disease improvement will ultimately be mediated by enhanced T cell activity. In some patients, overcoming T cell inhibition may be the only factor that needs to be addressed for disease improvement. The parameters addressed in this immunogram model, as briefly outlined below, are also helpful for understanding the interactions between other solid cancers and the immune system.

Tumor foreignness: for example, it is reported that the outcome to anti-CTLA-4 blockade therapy correlates with increased tumor mutational burden (a measure of neoantigen load) [169].

General Immune status: this may include a study of changes in immune cells in peripheral blood [170].

Immune cell infiltration: chemokines CXCL9 and CXCL10 that recruit CD8+ effector T cells are part of a gene signature associated with improved outcome to PD-1 blockade [18,171,172].

Checkpoint molecules: molecules such as PD-1 and PD-L1 on tumor cells or immune cells present potent immunosupression in TMEs [173,174,175].

Soluble inhibitors: IDO, a soluble molecule produced by TAMS or pDC, interferes with anti-CTLA-4 antibody efficacy in mice [176].

Absence of inhibitory tumor metabolism: high serum lactate dehydrogenase concentrations correlate with poor outcome to anti-CTLA-4 and anti-PD-1 antibody immunotherapy [177].

Tumor sensitivity to immune effectors: Tumor cells have developed several immune evasion mechanisms, such as inactivation of antigen-presentation machinery [102]. Additionally, by epigenetic post-translational mechanisms, the TME can select for cancer cells that can downregulate the expression of some tumor antigens, which would normally be recognized by T cells [178].

Other factors in the TME that regulate communication between cancers and the immune system include many of the parameters outlined in the preceding text, such as the maturation level and function of DC, the density and immunosuppressive nature of TAMS, NK-cell activity, and the TME architecture (Figure 1).

## 9. Conclusions and Perspectives

A better understanding of the TME in HGSOC will reveal useful diagnostic and prognostic biomarkers, and advance the development of suitable bioassays for routine clinical use for the detection and diagnosis of this malignancy. With such a heterogenous disease and multiple immune and biochemical networks, success in diagnosing this disease and predicting outcome will require multiple biomarkers, and more sensitive and precise methods of imaging to detect early lesions.

Current tools used to study the TME involve the use of genomics to investigate gene-expression signatures in the tumors of HGSOC. Verkaak and colleagues described four different gene classifications in a study of ovarian tumors as differentiated, immunoreactive, mesenchymal, and proliferative [179]. By IHC, the immunoreactive group had increased T lymphocytes, whereas desmoplasia associated with infiltrating stromal cells was in the mesenchymal group. Patients in the immunoreactive group had the best survival outcome. Some tumors also exhibited more than one of the 4 gene clusters. Findings were validated on an independent dataset of 879 HGSOC-expression profiles. Additional information to survival outcome and platinum resistance rates was obtained by using survival-outcome prediction models for association with BRCA1/2 mutation status, residual disease after surgery and stage of disease [179]. Similar gene-classification models may be useful for the selection of patients for targeted or immunotherapy, or to predict patient outcome. It is likely that patients exhibiting mesenchymal signatures may respond better to treatments such as angiogenesis inhibitors.

Additional methods to study the HGSOC TME include combinations of proteomic and other genomic data output [180,181] and a study approach addressing multiple parameters (such as gene expression, matrix proteomics, cytokine and chemokine expression, ECM parameters, and biomechanical properties) on a single biopsy sample for a better understanding of the events occurring in tumor tissue [182]. Other novel tools to study the ovarian TME include the use of artificial microenvironments to monitor ovarian-cancer progressiveness [183].

The HGSOC TME is a complex and dynamic interactive entity, which may vary between the primary disease and at the time of recurrence, and in the quest for more effective therapy design one needs to take into account pre-existing immunosuppression, as well as emerging resistance mechanisms with therapy [184]. Attempts to manage ovarian cancer with immunotherapy has not been as successful as for some other cancers [174,185]. We are hopeful that combining immunotherapy, such as PD-1 blockade, with other checkpoint inhibitory molecules (such as anti-CTLA-4, anti-TIM-3, anti-LAG-3), PARP inhibitors, kinase inhibitors, chemotherapeutics [186], dendritic-cell vaccines, CAR T cell therapy [187,188], or other treatments, will prove to be successful measures to overcome the multiple immunosuppressive mechanisms in the TME. As a cautionary measure, combination therapy will require optimizing doses and schedules of regimens, while limiting adverse effects. However, we anticipate that a combined therapy approach will be the way forward, towards providing effective therapy for improved survival, and ultimately a cure for HGSOC.

## Figures and Tables

**Table 1:** Phenotypic characterization of myeloid lineage cells in the ovarian TME.

Myeloid Group ^a^	Cell Classification	Phenotype
TAMS (monocytes/macrophages)	Inflammatory monocyte	CD14+, HLA-DR high, CD11c+, CD64+
	M1 macrophage	HLA-DR+, CD68+, CD80+, CD86+
	M2 macrophage	HLA-DR+, CD68+, CD163+, CD206+, CD200R
	M-MDSC	CD11b+, CD33+, CD14+, HLA-DR low
	G-MDSC	CD11b+, CD33+, CD15+, CD66b+, HLA-DR low
Dendritic cells	Immature DC	CD80 low, CD86 low, CD40 low, CD14+, CXCR3+
	Mature DC	C80 high, CD86 high, CD40 high, CD83+, HLA DR high, CCR7+, CD103+

**^a^** The primary identification markers of TAM subsets and of myeloid DC in the TME are shown.

**Table 2:** Immune dysregulation by TAMS in the TME.

Mediators ^a^	Cell Targets	Major Actions
IL-10	CTL	Inhibits activation
TGF-β	Treg	Induces differentiation
TGF-β, HGF, collagen, cathepsin and serine proteases, EGF, CSF-1	Tumor	Increases adhesion, invasion, and EMT
IL-6, TNF-α, WNT, JAG	Tumor	Promotes survival, growth, stemness
ADM, VEGF, COX-2, MMPs, HIF-1α, TGF-β	Endothelial	Angiogenesis

**^a^** TAMS elaborate a range of immune molecules and soluble mediators that are involved in the initiation and progression of cancer.
